# Identification of an antivirulence agent targeting the master regulator of virulence genes in *Staphylococcus aureus*

**DOI:** 10.3389/fcimb.2023.1268044

**Published:** 2023-10-31

**Authors:** Rekha Arya, Truc Kim, Joo Won Youn, Taeok Bae, Kyeong Kyu Kim

**Affiliations:** ^1^ Department of Precision Medicine, Sungkyunkwan University School of Medicine, Institute of Antibacterial Resistance Research and Therapeutics, Suwon, Republic of Korea; ^2^ Department of Orthopedic Surgery, University of Pittsburgh School of Medicine, Pittsburgh, PA, United States; ^3^ Department of Microbiology and Immunology, Indiana University School of Medicine-Northwest, Gary, IN, United States

**Keywords:** multiple drug-resistant *Staphylococcus aureus*, antimicrobial resistance, virulence gene expression, Staphylococcal accessory effector, antivirulence agent, SKKUCS

## Abstract

The emergence of bactericidal antibiotic-resistant strains has increased the demand for alternative therapeutic agents, such as antivirulence agents targeting the virulence regulators of pathogens. *Staphylococcus aureus* exoprotein expression (*sae*) locus, the master regulator of virulence gene expression in multiple drug-resistant *S. aureus*, is a promising therapeutic target. In this study, we screened a small-molecule library using a SaeRS green fluorescent protein (GFP)-reporter that responded to transcription controlled by the *sae* locus. We identified the compound, N-(2-methylcyclohexyl)-11-oxo-10,11-dihydrodibenzo[b,f][1,4]thiazepine-8-carboxamide (SKKUCS), as an efficient repressor of *sae*-regulated GFP activity. SKKUCS inhibited hemolysin production and reduced α-hemolysin-mediated cell lysis. Moreover, SKKUCS substantially reduced the expression levels of various virulence genes controlled by the master regulators, *sae*, and the accessory gene regulator (*agr*), demonstrating its potential as an antivirulence reagent targeting the key virulence regulators. Furthermore, autokinase inhibition assay and molecular docking suggest that SKKUCS inhibits the kinase activity of SaeS and potentially targets the active site of SaeS kinase, possibly inhibiting ATP binding. Next, we evaluated the efficacy and toxicity of SKKUCS *in vivo* using murine models of staphylococcal intraperitoneal and skin infections. Treatment with SKKUCS markedly increased animal survival and significantly decreased the bacterial burden in organs and skin lesion sizes. These findings highlight SKKUCS as a potential antivirulence drug for drug-resistant staphylococcal infections.

## 1 Introduction

The emergence of antimicrobial-resistant pathogens is a major challenge for drug development programs and poses a serious risk to public health (Baker et al., 2018; Zhang et al., 2021). *Staphylococcus aureus* is a prominent human pathogen that readily adapts to the host defense mechanisms against infections and causes various life-threatening diseases (Boldock et al., 2018; Kim et al., 2018). The increased incidence of community- and hospital-acquired *S. aureus* over the past decade is due to the rapid emergence of multiple drug-resistant isolates (Al-Mebairik et al., 2016; Rağbetli et al., 2016). Moreover, excessive use of antimicrobials leads to the emergence and spread of resistant strains, significantly compromising their efficacy (Hodille et al., 2017; Troeman et al., 2019). For instance, methicillin-resistant *S. aureus* is endemic to hospitals and community settings and exhibits resistance to various commonly used antibiotics, such as beta-lactams, glycopeptides, and fluoroquinolones. The emergence of vancomycin-resistant *S. aureus* strains has limited the efficacy of conventional antimicrobial treatments against staphylococcal infections, leading to increased morbidity and mortality worldwide (Panlilio et al., 1992; Appelbaum, 2006). Therefore, the development of alternative strategies is necessary to combat *S. aureus* resistance.

Recently, various approaches have been proposed to develop novel drugs for infectious bacterial diseases and improve the treatment efficacy. Of them, the most popular is antivirulence therapy, which interferes with bacterial virulence to prevent bacterial pathogens from invading the host cells and mitigates damage (Arya and Princy, 2013). Various global regulators have been proposed as drug targets because they control the expression of multiple genes involved in staphylococcal virulence functions, such as motility, biofilm formation, toxin production, and immune evasion (Arya et al., 2015; Chemmugil et al., 2019; Kwiecinski et al., 2021). For example, the accessory gene regulator (*agr*) system. which controls the expression of various virulence factors (Sully et al., 2014), has been a popular drug target to attenuate virulence and re-establish susceptibility to antimicrobials in drug-resistant *S. aureus* strains (Gov et al., 2001; Vasquez et al., 2017; Xie et al., 2020). However, the emergence of *agr*-negative clinical isolates in various infections, such as osteomyelitis, mastitis, and cystic fibrosis, limits the widespread application of *agr*-based therapies (Shopsin et al., 2008). To overcome the problems associated with *agr*-based therapeutic approaches, we have been exploring the *S. aureus* exoprotein expression (*sae*) system as an alternative target (Mizar et al., 2018; Yeo et al., 2018).

The *sae* locus encodes the SaeRS two-component signal transduction system composed of SaeP, Q, R, and S. SaeS is a sensor kinase in the cell membrane. Upon recognition of signals such as human neutrophil peptides (HNPs), it autophosphorylates H131 and subsequently transfers the phosphoryl group to SaeR. The phosphorylated SaeR, then, binds to and activates over 20 target promoters, most of which transcribe virulence genes. The *sae* locus has two promoters, P1 and P3. The P3 promoter is constitutive and transcribes only *saeS* and *saeR*, whereas the P1 promoter is autoinduced by Sae and transcribes the entire *saePQRS*. This locus is involved in the intricate regulation of various virulence genes (Steinhuber et al., 2003; Nygaard et al., 2010) and modulates the production of several key secretary virulence determinants, such as alpha-hemolysin (*hla*), gamma-hemolysin (*hlg*), coagulase (*coa*), Panton-Valentine leukocidin (*lukSF*), and fibronectin-binding proteins (*fnb*) (Steinhuber et al., 2003). As the *sae* locus is an excellent target for developing antivirulence agents, we aimed to investigate it further to facilitate the development of potent antivirulence agents. In this study, we screened a new chemical compound library, Korea Research Institute of Chemical Technology (KRICT), to identify a novel antivirulence compound that can treat staphylococcal infections.

## 2 Materials and methods

### 2.1 Ethics statement

All animal experiments adhered to the regulations of the Institutional Animal Care and Use Committee (IACUC). The study protocol was reviewed and approved by IACUC (SKKUIACUC2019-06-13-2), Sungkyunkwan University, School of Medicine. Mice (6–8-week-old) were purchased from Orient Bio Inc. (Seongnam, South Korea) and used for subsequent experiments.

### 2.2 Bacterial strains and growth conditions

*S. aureus* strains and plasmids used in this study are listed in **Supplementary Table S1**. Bacteria were grown in tryptic soy broth (TSB; Sigma-Aldrich, MO, USA). *Escherichia coli* was grown in Lysogeny broth (LB) (Sigma-Aldrich) or agar plates. The reporter strain was maintained in chloramphenicol at 5 µg/mL. The culture was incubated at 37°C with shaking (180 rpm), collected via centrifugation, and washed with sterile phosphate-buffered saline (PBS; Sigma-Aldrich, MO, USA) for injection. The number of viable bacteria was determined after serial dilutions and plating on blood agar (**Supplementary Table S1**).

### 2.3 Construction of GFP-promoter fusion (reporter strain)

Reporter plasmid pCL-P*_hlamin_-gfp* was generated using a ligation-independent cloning method, as previously described (Donnelly et al., 2006; Yeo et al., 2018). Briefly, pCL55-*gfp* was used for vector polymerase chain reaction (PCR) with P1969 and P1747 primers (Cho et al., 2015). P1971 and P1972 primers were used to amplify the DNA sequence containing the promoter site. T4 DNA ligase was used to treat the PCR products in the presence of dGTP (insert DNA) or dCTP (vector). Next, *E. coli* DH5α was used for the transformation of the mixed products. After verification, all plasmids were electroporated into *S. aureus* strain RN4220 and subsequently transduced into *S. aureus* USA300 using ϕ85.

### 2.4 Screening of the KRICT chemical library

A library of nearly 1,000 chemical products was purchased from KRICT. All compounds were 80% pure; they were dissolved in dimethyl sulfoxide (DMSO; Sigma, USA) and used without any further purification. Drug screening was performed as follows: GFP reporter strain was used for high-throughput screening to identify the antimicrobial and antivirulence potencies of library compounds. GFP reporter strain was collected from TSA plates and suspended in fresh TSB medium. Next, the cultures were incubated at 37°C in a rotary shaker at 180 rpm and allowed to grow to the exponential phase, and cell density was adjusted from 1 × 10^5^ to 5 × 10^5^ CFU/mL. The cultures were added to a 48-well plate (Nunc, Wiesbaden, Germany) with 10 µM of KRICT chemicals and incubated for 8 h. Induced fluorescence of the reporter strain was compared between the vehicle controls and compounds in 0.1% DMSO, and the optical density at 600 nm and fluorescence at 480/520 nm were measured (Biotek, Vermont, USA). Next, the selected lead concentrations were used in a two-fold serial dilution to inhibit the GFP expression by 50% (IC_50_) and 90% (IC_90_) compared to that in the vehicle control. The calculated IC_50_ and IC_90_ values were used in all subsequent experiments to analyze the efficacy of the selected lead compound.

### 2.5 Bacterial growth assay

GFP strains were grown overnight in TSB and diluted 1:100 in fresh TSB to reach an early exponential growth phase. Then, 5 × 10^5^ cells were seeded in a 48-well polystyrene plate (Merck, Darmstadt, Germany) without or with effective concentrations of SKKUCS (IC_50_ and IC_90_ values of 5 and 10 µM, respectively). The cells were grown at 180 rpm for 10 h at 37°C, and the optical density was measured at 600 nm and compared with that of the vehicle.

### 2.6 Hemolytic activity assay

Hemolytic activity of SKKUCS was determined with sheep erythrocytes (Icell, South Korea). *S. aureus* USA300 was cultured overnight, diluted to 5 × 10^5^ CFU, and incubated with SKKUCS for 8 hr at 37°C and 180 rpm. The supernatants were collected for further analysis. Then, 5% sheep erythrocytes were prepared by centrifuging 1 mL fresh sheep blood (1620 × g, 10 min) and resuspending the pellet in 1 mL sterile PBS. Culture supernatant with or without SKKUCS (100 µL) were mixed with 100 µL of 5% red blood cells and incubated at 37°C for 30 min. Samples were centrifuged (Eppendorf, Hamburg, Germany) at 1000 rpm, and hemolysis was determined by measuring the optical density of the supernatant at 540 nm with an ELISA plate reader (Biotek, USA) (Tal-Gan et al., 2013; Arya et al., 2015).

### 2.7 Quantitative reverse transcription-PCR analysis

qRT-PCR was performed for transcripts of interest relative to 16S rRNA using an intercalating dye-based method as previously described, with minor modifications (Mizar et al., 2018). *S. aureus* USA300 cultures were treated with SKKUCS and incubated for 6 h. Total RNA was isolated and purified with the Qiagen RNA Protect Bacteria Reagent and RNeasy Mini Kit (Qiagen; Valencia, CA) via enzymatic disruption. RNA was further purified with silica columns, treated with DNase, and reverse-transcribed into cDNA with a cDNA reverse transcription kit (Random Hexamer; Clontech). qPCR was performed using a CFX Connect Real-Time System (Bio-Rad; Hercules, CA, USA) and SYBR Green PCR Master Mix (Bio-Rad). All samples were assayed in triplicate and normalized to 16S rRNA (**Supplementary Table S2**) (Park et al., 2007; Jin et al., 2020).

### 2.8 Cell viability assay

HeLa cells were obtained from the American type culture collection (ATCC; ATCC CCL-2) and propagated in high-glucose Dulbecco’s modified Eagle’s medium (DMEM) supplemented with 10% fetal bovine serum (FBS; Invitrogen, California, USA) and 1% penicillin/streptomycin in a 5% CO_2_ incubator. Cells were seeded in glass-bottomed dishes (Nunc, USA) at 1 × 10^6^ cells and incubated with SKKUCS (concentration equivalent to IC_50_ and IC_90_) for 1 h prior to infection (Stelzner et al., 2021). *S. aureus* USA300 was grown overnight and centrifuged at 4000 rpm for 10 min. The cells were washed with sterile PBS and adjusted to OD_600 =_1. HeLa cells were infected with USA300 at a multiplicity of infection (MOI) of 1:10 for 45 min. The cells were washed with PBS and then treated with fluorescein diacetate and propidium iodide for 30 min at room temperature in darkness. The cells were washed again with PBS, and fluorescence images were acquired with a confocal microscope (Carl Zeiss Microscopy, Germany) (Wang et al., 2020).

### 2.9 Invasion assay

RAW264.7 cells (ATCC TIB-71) were used for invasion assays. Briefly, RAW264.7 cells were grown in a high-glucose DMEM medium containing 10% FBS at 37°C with CO_2_. When the cells reached 70% confluency, 5 × 10^5^ cells were seeded into a 24-well plate and incubated for 48 h. *S. aureus* USA300 was cultured overnight in TSB and centrifuged at 4000 rpm for 10 min (Zang et al., 2020). The pellet was washed thrice and resuspended in PBS to reach an OD_600_ of 1, corresponding to 10^9^ CFU/mL. IC_50_, IC_90_, and 2xIC_90_ doses of SKKUCS were added to the wells 1 h prior to infection. USA300 was applied to RAW264.7 cells at MOI 1:10 and incubated at 37°C with CO_2_ for 45 min. The cells were washed, and gentamicin (100 µg/mL) was added to kill the extracellular or adherent staphylococci. After 60 min incubation, the cells were washed with PBS, and the internalized bacteria were counted by spreading them on blood agar (BAP) plates. The plates were incubated at 37°C for 18 h, and the colonies were counted (Gunaratnam et al., 2019).

### 2.10 *In vitro* phosphorylation assay

We expressed and purified the cytoplasmic kinase domain of *S. aureus* SaeS (amino acids 118–351) and carried out the *in vitro* phosphorylation assay, as we reported previously (Mizar et al., 2018) with a minor modification. Briefly, purified SaeS (5 µM) was incubated with each test compound (50 µM) or 10% DMSO (vehicle control) in the reaction buffer on ice for 15 min. Then, the mix of ATP and [γ-^32^P]ATP (total phosphate donor concentration of 130 µM) was added to initiate the autokinase reaction. The reactions were subjected to SDS-PAGE, and the polyacrylamide gel was further dried on a filter paper and exposed to an X-ray film to visualize the phosphorylated SaeS bands. The phosphorylation levels of SaeS were then quantified by densitometric analysis, and its autokinase activities were calculated as the percentages of phosphorylated protein in the samples challenged with the tested compounds compared to that in the sample challenged with 10% DMSO (vehicle control).

### 2.11 Molecular docking study

A structural atomic model of the sensor histidine kinase SaeS from *S. aureus* USA300 was collected from an AlphaFold database (Varadi et al., 2022) with an AlphaFoldDB ID of Q2FIT5. The chemical structures of ATP and SKKUCS were obtained from an NCBI PubChem database (Kim et al., 2023) with their PubChem CIDs of 5957 and 4167768, respectively, and were prepared using Open Babel (O’Boyle et al., 2011) prior to the docking study. The *in silico* molecular dockings of ATP and SKKUCS into the kinase domain of *S. aureus* SaeS (residues 118–351) were performed using GNINA (McNutt et al., 2021), which can be accessed via an executable Google Collaboration notebook provided at https://github.com/gnina/gnina. To validate the affinity scores derived from GNINA, we estimated the binding affinity of each docking pose of ATP and SKKUCS to SaeS using HAC-Net (Kyro et al., 2023), which can be accessed via an executable Google Collaboration notebook provided at https://github.com/gregory-kyro/HAC-Net.

### 2.12 Animal survival assay after intraperitoneal infection

*S. aureus* USA300 was grown overnight in TSB. The next day, the culture was diluted 1:100 in fresh TSB medium and allowed to grow to an early exponential growth phase. The culture pellet was washed with DPBS to reach 5 × 10^7^ CFU for the infection. The cells were injected intraperitoneally with 50 µL of DPBS into mice. DMSO was used as the negative control. Mice were treated with various doses of SKKUCS or vehicle at 12 h post-infection. The mice were administered SKKUCS via the tail vein every 12 h for one week. The mice were monitored daily, and weight loss was recorded. One-week post-infection, the mice were euthanized via CO_2_ inhalation, and the kidney, spleen, liver, and heart were excised. The samples were placed in 1 mL of DPBS for homogenization, and the CFU were counted in BAP (Yeo et al., 2018).

### 2.13 Establishment of the dermonecrosis infection model

For the skin infection experiment, SKKUCS was solubilized at 1 mg/mL in DMSO (Sigma) and diluted further in DPBS for mice injection. DMSO was used as the negative control. BALB/c mice (6–8-week-old, 20–25 g, female) were obtained from Orient Bio Inc (Korea). The lower backs of the mice were shaved, and chemical depilatories were applied. An overnight culture of *S. aureus* USA300 was diluted 100-fold into fresh TSB and further incubated for 4 h at 37 °C. The bacterial culture was washed with DPBS and prepared 1 ×10^7^ for infection. The mice were subsequently treated with SKKUCS at 8 h post-infection. The first group of infected mice was subcutaneously treated with 5 µM, the second group with 10 µM, and the third group with 20 µM. The final doses of SKKUCS were 109.9, 219.8, and 439.7 µg/kg, respectively. The chemical was repeatedly administered to the mice for one week at every 12 h interval. The lesion area was measured with calipers as previously described (Cheung et al., 2011; Dokoshi et al., 2020) and recorded daily in addition to body weight loss. The ulcer area (mm^2^) was calculated using the following equation: (π/2)([length of the abscess] × [width of the abscess]). After one week, the mice were euthanized via CO_2_ inhalation. The ulcer area was excised, and the spleen was removed. The samples were placed in 1 mL DPBS and homogenized for bacterial CFU count. The samples were diluted and plated on blood agar as previously described.

### 2.14 Statistical analyses

Statistical analyses were conducted using the GraphPad Prism software (version 7.0). Student’s *t*-test was used to compare the two datasets. One-way ANOVA analysis of variance was used for more than three datasets. Log-rank test (Mantel–Cox) was used for survival analysis. A non-parametric Mann–Whitney test (two-tailed) was used to compare the two groups. Statistical significance was set at *p* < 0.05.

## 3 Results

### 3.1 Small molecule library screening for Sae inhibitor

**Figure 1:**
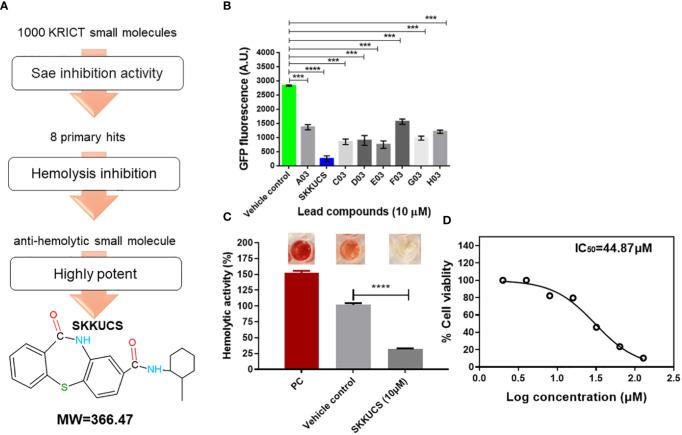
N-(2-methylcyclohexyl)-11-oxo-10, 11-dihydrodibenzo[b,f][1,4]thiazepine-8-carboxamide (SKKUCS) suppresses green fluorescent protein (GFP) expression in USA300P*_hlamin_* strain. **(A)** Schematic representation of the screening procedure. **(B)** Potent leads from the Korea Research Institute of Chemical Technology (KRICT) used to reduce GFP expression in USA300-P*_hlamin_-gfp* were analyzed at 8 h. **(C)** The most potent lead was further tested for hemolysis inhibition and analyzed at 8 h. **(D)** HeLa cells were seeded, treated with SKKUCS (2–128 µM), and incubated for 72 h. Lactate dehydrogenase (LDH) release was measured at OD_450nm_. All experiments were performed in triplicate, and significance was compared to the vehicle. Statistical significance was measured using an unpaired two-tailed Student’s t-test. ***p < 0.001; ****p<0.0001.

An overview of the primary screening of antivirulence agents is presented in **Figure 1A**. The α-hemolysin (*hla)* gene is a well-characterized target of the SaeRS TCS, and its promoter has the SaeR-binding sequence (Nygaard et al., 2010). Therefore, in this study, we decided to screen the KRICT chemical library using a previously constructed SaeRS reporter strain (Mizar et al., 2018; Yeo et al., 2018). We generated a minimal P *hla*, termed P*_hlamin_*, containing only the SaeR-binding site and the –35 and –10 promoter sequences. P*_hlam_* was fused with the green fluorescent protein (*gfp*) gene in a single-copy integration vector, pCL55, and the resulting plasmid, pCL-P*_hlamin_-gfp*, was inserted into *S. aureus* USA300. The resulting SaeRSGFP reporter strain was used to screen approximately 1000 small molecules from the KRICT library to identify eight compounds that repressed GFP expression by more than 50% at 10 μM. These were considered primary hits (**Figure 1B**). All primary hits were assessed for their anti-hemolysis activity, and only one molecule exhibited a sharp reduction in hemolysis (**Figure 1C**). The molecule, N-(2-methylcyclohexyl)-11-oxo-10,11-dihydrodibenzo[b,f][1,4]thiazepine-8-carboxamide (M.W. 366.5), was named SKKUCS and further studied. With USA300-P*_hlamin_*-*gfp*, SKKUCS showed a half-maximal inhibitory concentration (IC_50_) of 5 μM and a 90 percent inhibitory concentration (IC_90_) of 10 μM. At IC_90_, SKKUCS exhibited a minimal growth inhibitory effect on USA300 (**Supplementary Figure S1**).

Next, we measured the cytotoxicity of SKKUCS with HeLa cells. using the lactate dehydrogenase (LDH) release assay. HeLa cells were incubated with various concentrations of SKKUCS (2–128 μM), and the release of lactate dehydrogenase (LDH) was measured by monitoring the optical density at 450 nm. SKKUCS showed a mild cytotoxicity to HeLa cells with IC_50_ of 44.87 μM (**Figure 1D**).

### 3.2 Inhibitory effects of SKKUCS on SaeRS GFP reporter and hemolysis

**Figure 2:**
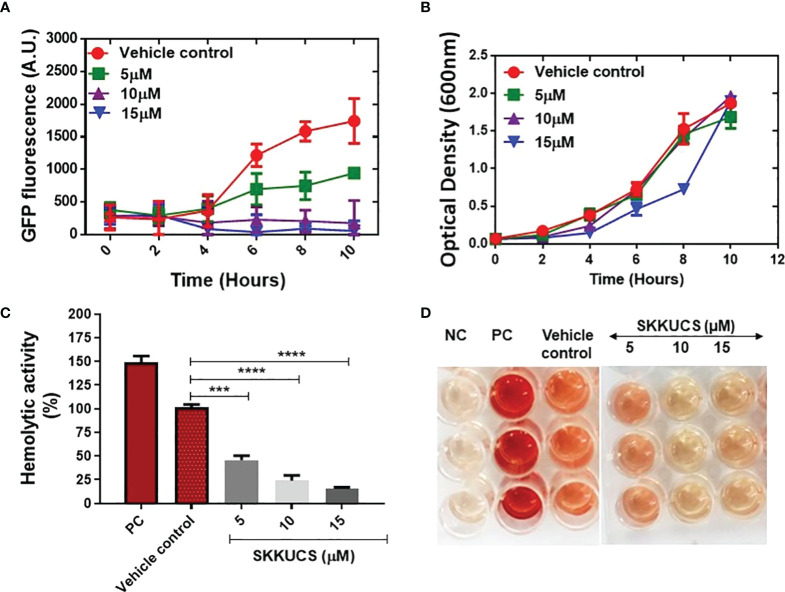
Effects of SKKUCS on *Staphylococcus aureus* growth and hemolysis inhibition. **(A)** USA300-P*_hlamin_-gfp* was grown with or without SKKUCS (5–15 µM) as vehicle control for 10 h. **(B)** Cell growth was measured in a time-dependent manner with various concentrations of SKKUCS. **(C)** Various concentrations of SKKUCS were used to treat sheep red blood cells (RBCs), and percentage hemolysis was calculated relative to the vehicle control. **(D)** Qualitative hemolysis. All experiments were performed in triplicates and significance was compared to the control (no treatment) using one-way analysis of variance (ANOVA). NC, negative control; PC, positive control. ***p<0.001,****p<0.0001.

Next, we evaluated the antivirulence effects of SKKUCS. SaeRS GFP reporter was incubated with various concentrations of SKKUCS (5-15µM), and the fluorescence signal was measured at different time points. As shown in **Figure 2A**, incubation of GFP-reporter with the IC_50_ and IC_90_ concentrations of SKKUCS reduced the *gfp* expression by 48.12 ± 3.9 and 82.4 ± 1.6%, respectively. In addition, incubation with 15 µM SKKUCS for 10 h decreased the fluorescence intensity by 93.03% (**Figure 2A**). As we aimed to develop a novel SaeRS-based inhibitor specifically targeting the *S. aureus* virulence without affecting its growth, we measured the inhibitory effect of SKKUCS on the growth of *S. aureus* USA300 in a time-dependent manner. SKKUCS showed no significant growth-inhibitory effect up to 10 μM, and showed small growth inhibition at 15 µM (**Figure 2B**).

SaeR binds to the promoter region of *hla* and activates its expression, which plays a critical role in erythrocyte hemolysis during staphylococcal infections. Here, we tested the effect of SKKUCS on erythrocyte lysis by *S. aureus* USA300. As shown in **Figures 2C, D** the IC_50_ and IC_90_ concentrations of SKKUCS reduced the hemolytic activity of USA300 by 45.7 ± 4.7 and 76.0 ± 5.4%, respectively, as compared to the hemolysis observed in non-treated samples. Additionally, hemolysis was inhibited by 85.5 ± 3.5% when the samples were treated with 15 μM SKKUCS. These results are consistent with the IC_90_ values observed for the SaeRS-GFP reporter strain. Time-dependent assay further demonstrated that SKKUCS had no impact on the growth curve of *S. aureus* USA300 compared to that of the control at various concentrations.

### 3.3 Effects of SKKUCS on virulence gene expression

**Figure 3:**
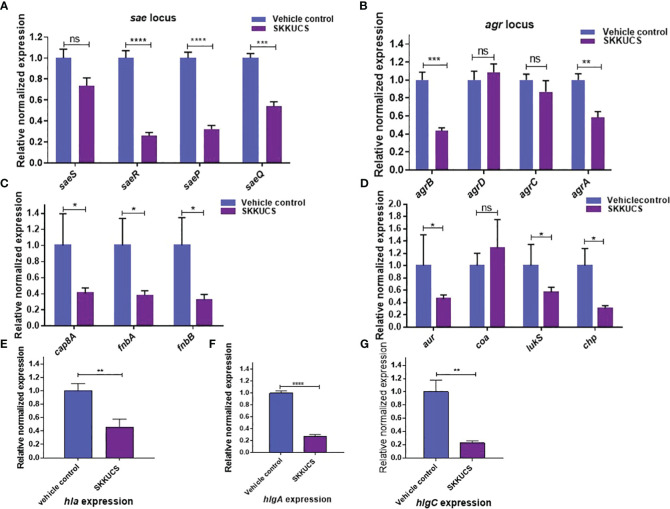
Effects of SKKUCS on virulence gene expression. *S. aureus* USA300 was treated with SKKUCS (10 µM) for 6 h, and the transcription levels of virulence genes were analyzed. **(A)** Staphylococcal accessory effector (*sae*) locus gene expression. **(B)** Accessory gene regulator (*agr*) locus gene expression. **(C)** Genes involved in adherence: capsular polysaccharide (*cap8A*), fibronectin-binding proteins (*fnbA* and *fnbB*). **(D)** Aureolysin (*aur*), coagulase (*coa*), leukocidins (*lukS*), and chemotaxis inhibitory protein (*chp*). **(E)** α-hemolysin (*hla*). **(F)** ϒ-hemolysin component A (*hlgA*). **(G)** ϒ-hemolysin component C (*hlgC*). The Y-axis indicates the mRNA level relative to 16S rRNA, whose transcription was not significantly affected by SKKUCS. All experiments were performed in triplicate, and statistical significance was measured using an unpaired two-tailed Student’s t-test. *p < 0.05; **p < 0.01; ***p < 0.001; ****p<0.0001; ns, not significant.

We further analyzed the effects of SKKUCS on the transcription of virulence genes. In this analysis, we used 10 µM of SKKUCS, the IC_90_ of the SaeRS GFP reporter. SKKUCS suppressed the transcription of the *sae* operon, except for *saeS* (**Figure 3A**). Since the SaeRS TCS is required for the expression of *hla* (Gudeta et al., 2019), the suppression of the *sae* transcription explains the inhibitory effect of SKKUCS on the hemolysin activity (**Figures 2C, D**). Agr is another global regulator required for the *hla* expression. The *agr* locus comprises four regulatory genes, of which *agrA* and *agrC* constitute a two-component system, whereas *agrB* encodes a transmembrane protein required for the processing of the signal pre-peptide, AgrD. As shown in **Figure 3B**, SKKUCS suppressed the transcription of *agrA* and *agrB*, raising the possibility that SKKUCS suppresses the Agr system too. To confirm the suppression of the SaeRS TCS, we examine the effects of SKKUCS on the transcription of the Sae regulons. As shown in **Figures 3C–G**, the transcripton of the Sae regulons, except for *coa*, was reduced by SKKUCS, further confirming the Sae-suppressive activity of SKKUCS.

### 3.4 SKKUCS prevents *S. aureus*-mediated cellular damage

**Figure 4:**
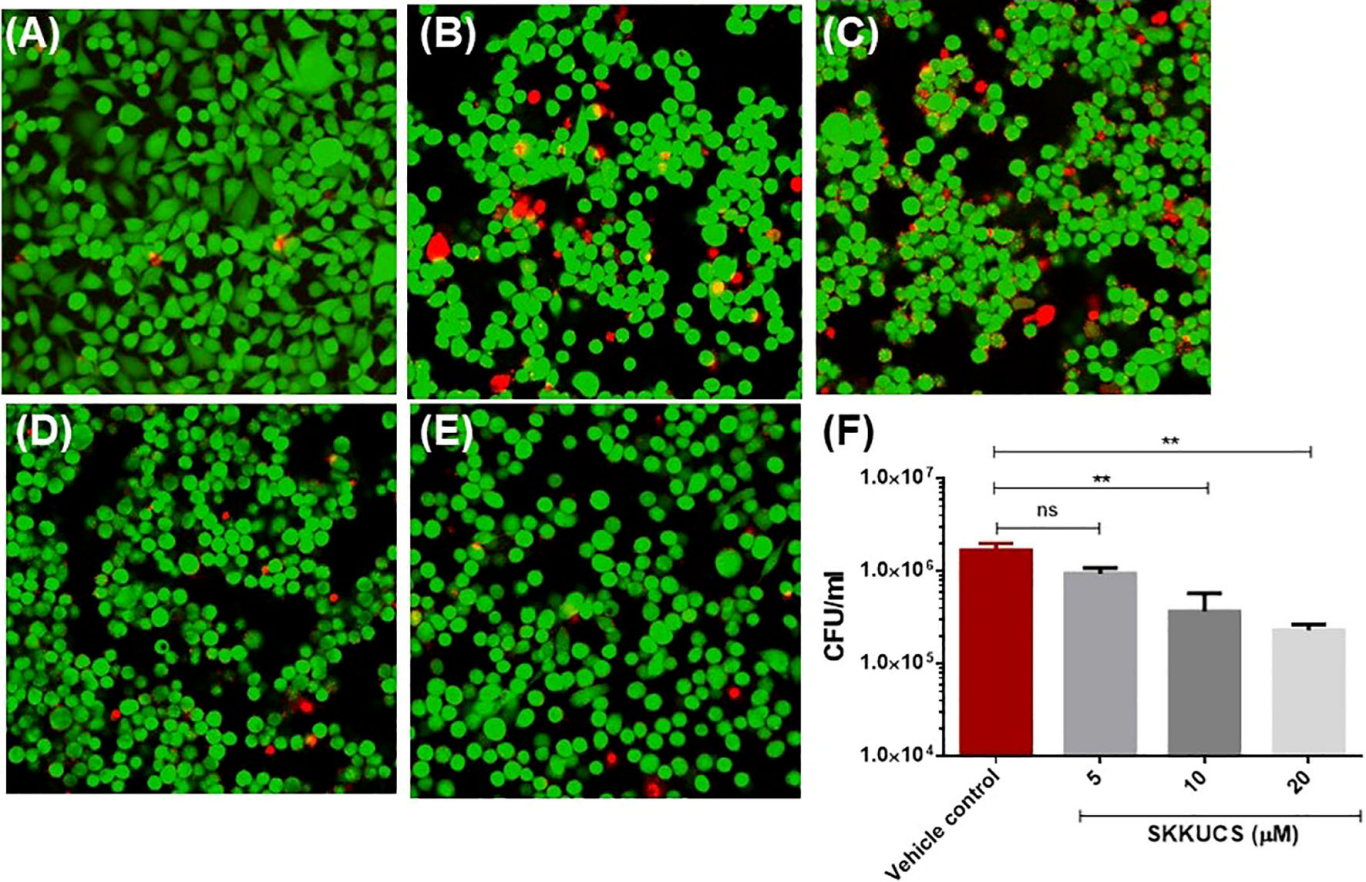
SKKUCS protects HeLa cells from α-hemolysin-mediated cell damage and reduces the intracellular survival of *S. aureus* USA300. HeLa cells were seeded in 1 x 10^6^ and treated with SKKUCS. HeLa cells were infected with *S. aureus* USA300 at 1:10 multiplicity of infection (MOI) and incubated for 45 min. Then, cells were stained with fluorescein diacetate (FDA) and propidium iodide (PI) and imaged under the confocal microscope. Green, live; red, dead, **(A)** no infection; **(B)** vehicle control; **(C)** 5 µM; **(D)** 10 µM; **(E)** 20 µM. **(F)** RAW264.7 cells were seeded in 1 x 10^6^ CFU and treated with SKKUCS. RAW264.7 cells were infected with *S. aureus* USA300 at 1:10 MOI and incubated for 45 min. Extracellular *S. aureus* was lysed with gentamicin (100 µg/mL), and internalized *S. aureus* in blood agar plates was counted. All experiments were performed in triplicate, and statistical significance was measured using an unpaired two-tailed Student’s t-test. **p < 0.01; ns, not significant.

HeLa cells are widely used as a model to explore the cellular responses to pore-forming cytotoxins (Thay et al., 2013). α-hemolysin is well known for its cytotoxicity (Vandenesch et al., 2012; Thay et al., 2013). Therefore, we examined whether SKKUCS can protect HeLa cells from damages induced by α-hemolysin from *S. aureus* USA300. Hela cells were infected with USA300 in the presence of various concentrations of SKKUCS. Then, the HeLa cells were subjected to Live/Dead staining. Based on the image analysis presented in **Figure 4C**, it is clear that SKKUCS at IC_50_ concentration has minimal impact on cell viability as compared to the control. In the control group, approximately 22% of HeLa cells infected with USA300 were detected as dead cells and compared with no infection control (**Figures 4A, B**), whereas only 19% of cell death was observed in the IC_50_-treated group (**Figure 4C**). However, when USA300 infected HeLa cells were treated with IC_90_ or 2xIC_90_, the number of dead cells decreased significantly to 3.5% and 2.6%, respectively (**Figures 4D, E**). This indicates that SKKUCS was able to protect HeLa cells at these concentrations from damage, in comparison to the vehicle control group (**Supplementary Figure S3**).

Next, we investigated the effects of SKKUCS on the intracellular survival of USA300 in macrophages (RAW264.7 cells). The SKKUCS treatment at IC_50_ and IC_90_ reduced the intracellular survival of USA300 by 41.0 and 78.1%, respectively (**Figure 4F**). When the concentration was increased to 2×IC_90_, SKKUCS reduced the intracellular survival of USA300 by 85.91% (**Figure 4F**).

### 3.5 SKKUCS inhibits histidine autokinase activity and interacts with SaeS

**Figure 5:**
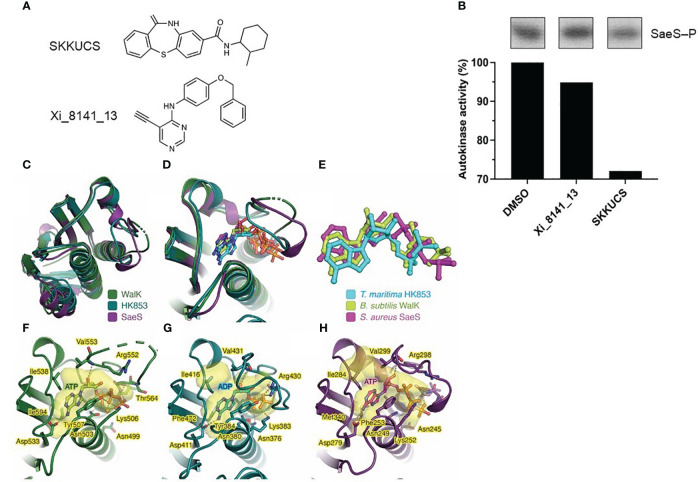
Inhibitory effect of SKKUCS on the autokinase activity of SaeS and molecular docking. **(A)** Chemical structures of compounds tested for their inhibitory effects on the *in vitro* autokinase of SaeS. **(B)** Autophosphorylations of SaeS in the presence of different tested compounds. Purified recombinant SaeS (5 µM) was incubated with either SKKUCS or Xi_8141_13 (50 μM each) for 15 min prior to the addition of ATP (130 μM) to initiate the autophosphorylation reactions. Following SDS-PAGE analysis, phosphorylated SaeS were visualized by autoradiography and quantified by densitometric analysis. The autokinase activities were calculated as the percentages of phosphorylated proteins in the samples challenged with the tested compounds compared to that in the sample challenged with 10% DMSO (vehicle control). **(C)** Structural superimposition of the ATP-binding domains of *S. aureus* SaeS (AlphaFoldDB: Q2FIT5), *B. subtilis* WalK (PDB: 3SL2), and *T. maritima* HK853 (PDB: 6RGZ). **(D)** Structural alignment as in **(C)**, focused on the ATP-binding sites with the bound nucleotides. **(E)** Top-scored docking pose of ATP within SaeS in superimposition with experimentally determined ADP bound to HK853 and ATP bound to WalK. **(F–H)** Protein residues interacting with nucleotides observed in **(F)** *B. subtilis* WalK, **(E)** *T. maritima* HK853, and **(G)** *S. aureus* SaeS. Each protein structure is shown as a cartoon representation. Protein cavities accommodating the nucleotide substrate were calculated using PyVOL (Smith et al., 2019) and are represented as transparent surfaces. The bound nucleotide in each structure and the protein residues involved in nucleotide interactions are shown as stick models. Hydrogen and ionic bonds are drawn as yellow dashed lines connecting interacting atoms. Figures were prepared using PyMOL (http://www.pymol.org).

The kinase activity of SaeS is essential for the virulence of *S. aureus* (Liu et al., 2015). It is possible that SKKUCS exerts its antivirulence activity by interfering with the SaeS kinase activity. To test the possibility, we examined the effect of SKKUCS and the control compound xi_8141_13 on the autophosphorylation activity of the recombinant SaeS kinase domain (residues 118–351) (**Figure 5A**). The SaeS phosphorylation was slightly reduced by Xi_8141_13 compared to DMSO; however, it was reduced approximately by 30% by SKKUCS (**Figure 5B**). To further understand the inhibitory effect of SKKUCS on the autokinase activity of SaeS, we carried out in silico molecular docking experiment. Due to the unavailability of an experimentally resolved structure of *S. aureus* SaeS, we employed an AlphaFold-derived atomic model of the kinase domain of SaeS for the docking study. A structural similarity search using FoldSeek (van Kempen et al., 2023) unveiled a striking resemblance between the C-terminal catalytic and ATP-binding domain of SaeS and those of crystallographically resolved structures of *Thermotoga maritima* HK853 complexed with ADP (Mideros-Mora et al., 2020) and *Bacillus subtilis* WalK complexed with ATP (Celikel et al., 2012), indicating a nucleotide-bound state of the AlphaFold model of SaeS (**Figure 5C**).

Supporting this observation, molecular docking of ATP into the kinase domain of SaeS revealed that the top-scored docking pose of ATP fitted well to the ATP-binding pocket SaeS (**Figure 5D**). Remarkably, the conformation of the docked ATP closely resembled those of other nucleotides occupying the similar pockets in WalK and HK853 (**Figure 5E**). Moreover, SaeS residues implicated in accommodating the docked ATP are similar to those observed in the crystal complex structures of WalK-ATP and HK853-ADP (**Figures 5F–H**). Collectively, the *in silico* molecular docking study exhibited its efficacy in accurately determining the conformation of ATP within SaeS and thus enabled us to identify the binding site of SKKUCS in the SaeS structure.

The molecular docking of SKKUCS into the kinase domain of SaeS revealed that all three top-scored docking poses of this chemical not only accommodated well to the ATP-binding pocket but also superimposed well to the ATP molecule (**Supplementary Figure S2**). Consistently observed in these docking poses, the SKKUCS’s dibenzothiazepine moieties likely competed with the adenine moiety of ATP, while their methylcyclohexane moieties likely competed with the ATP’s phosphate groups. Due to the predominance of ring structures in SKKUCS, it is conceivable that hydrophobic interactions primarily drive the binding of this chemical within the ATP-binding pocket of SaeS (**Supplementary Figure S2**). In this pocket, SKKUCS probably shared certain interacting residues with ATP, most notably Phe253 and Arg298 (**Figure 5H**; **Supplementary Figure S2**).

### 3.6 Validation of the antivirulence activity of SKKUCS using the mouse peritoneal infection model

**Figure 6:**
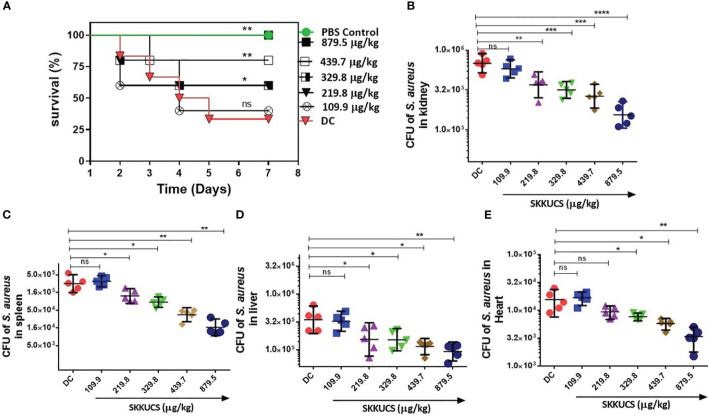
Dose-dependent efficacy of SKKUCS in a peritoneal infection model. *S. aureus* USA300 (5 x 10^7^ CFU) was intraperitoneally (i.p.) injected into mice. At 8 h post-infection, SKKUCS (109.9, 219.8, 329.8, 439.7, and 879.5 µg/kg body weight) was injected into the mice via the tail vein. The doses were provided at 12 h intervals for seven days. **(A)** Seven days post-infection, the percentage survival was calculated as compared to the vehicle. **(B)** Bacterial burden in the kidney at day 7. **(C)** Bacterial burden in the spleen at day 7. **(D)** Bacterial burden in the liver at day 7. **(E)** Bacterial burden in the heart on day 7. All experiments were performed using biological replicate samples (n=5). Statistical significance was calculated using the Log-rank test. ****p<0.0001, ***p < 0.001, **p < 0.01, *p < 0.05 and ns, not significant via the Student’s t-test.

Next, we further validated the antivirulence activity of SKKUCS in a mouse model. The mice were challenged with 5 × 10^7^ colony forming units (CFUs) *S. aureus* USA300 via an intraperitoneal (i.p.) injection and treated with SKKUCS at IC_50_ and IC_90_ doses (109.9 and 219.8 µg/kg body weight, respectively) via the tail vein 8 hrs post-infection. Drugs were administered to the mice every 12 hrs for one week. Compared to the no-drug control, IC_50_ and IC_90_ doses of SKKUCS protected the mice and increased their survival by 40 and 60%, respectively (**Figure 6A**). To assess the efficacy of SKKUCS at higher doses, we increased the doses of SKKUCS from 329.8 to 879.5 µg/kg body weight. SKKUCS administered at a dosage of 329.8 µg/kg demonstrated comparable efficacy as IC_90_ and protected the mice up to 60% when compared to the disease control group. High doses of 439.7 and 879.5 µg/kg SKKUCS significantly increased the survival of animals to 80 and 100%, respectively, compared to that in the disease control. To further investigate the potency of SKKUCS, we examined the bacterial load in a murine intraperitoneal infection model one-week post-infection with USA300. According to the data presented in **Figure 6B**, the bacterial burden in the kidney decreased progressively in response to the increasing doses. IC_90_ dose of SKKUCS reduced the *S. aureus* USA300 burden to 45.6% (3.8 × 10^5^ CFU/mL), and its higher dose (879.5 µg/kg) reduced the bacterial count by 77.0% (1.6 × 10^5^ CFU/mL). Next, bacterial burdens in the spleen, liver, and heart were calculated and compared with those in the no-drug control. SKKUCS significantly reduced the bacterial counts in a dose-dependent manner. IC_90_ dose of SKKUCS reduced the bacterial count to 56.5% (1.3 × 10^5^ CFU/mL) in the spleen (**Figure 6C**), 54.1% (1.7 × 10^5^ CFU/mL) in the liver (**Figure 6D**), and 39.7% (9.4 × 10^3^ CFU/mL) in the heart (**Figure 6E**). A higher dose of SKKUCS (879.5 µg/kg) significantly reduced the bacterial burden in the spleen, liver, and heart by 94.2% (1.3 × 10^4^ CFU/mL), 74.3% (9.6 × 10^4^ CFU/mL), and 78.37% (3.3 × 10^3^ CFU/mL), respectively, compared to that in the disease control.

### 3.7 SKKUCS treatment *in vivo* attenuates tissue injury

**Figure 7:**
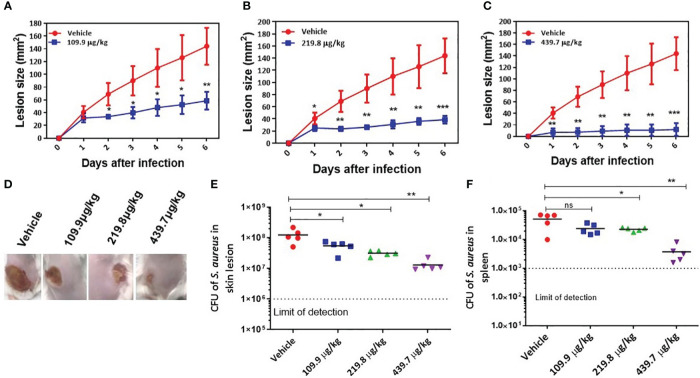
*In vivo* efficacy of SKKUCS in attenuating the dermonecrosis infection. *S. aureus* USA300 (1 x 10^7^ CFU) was injected subcutaneously into the flank (n=5 mice per group) of BALB/c mice. The mice were treated with SKKUCS (109.9, 219.8, and 439.7 µg/kg of body weight) or vehicle 12 h post-infection. The mice were treated with SKKUCS at 12 h intervals for one week. Parameters show the lesion development and bacterial burden in the lesion site and spleen. **(A)** Lesion formation in the group treated with 109.9 µg/kg SKKUCS. **(B)** Lesion formation in the group treated with 219.8 µg/kg SKKUCS. **(C)** Lesion formation in the group treated with 439.7 µg/kg SKKUCS. **(D)** Lesion images were taken one-week post-infection. **(E, F)** Bacterial burden was calculated in the skin lesion and spleen one-week post-infection. All data from mouse infection were represented as the mean ± standard error of the mean (SEM) ***p < 0.001, **p < 0.01, *p < 0.05 and ns, not significant via the Student’s t- and two-tailed Mann–Whitney U tests.

*S. aureus* is a common cause of skin and soft tissue infection (Moran et al., 2006). Therefore, we further investigated the anti-virulence activity of SKKUCS in an *in vivo* skin infection model. Mice were challenged with *S. aureus* USA300 (1 × 10^7^ CFU) via an intradermal route. At 8 h post-infection, the mice were treated with 109.9 µg (IC_50_), 219.8 µg (IC_90_), or 439.7 µg/kg (2xIC_90_) body weight. SKKUCS was intradermally administered to the mice every 12 h for one week. Mice infected with USA300 without any treatment (vehicle) developed skin ulcers within two days, reaching a maximum area of 144.0 ± 31.6 mm^2^. During the same period, each group treated with the IC_50_ dose of SKKUCS had considerably reduced lesion size, reaching a maximum of 58.8 ± 13.8 mm^2^ (**Figure 7A**). Treatment with the IC_90_ dose of SKKUCS further reduced the lesion size to 38.8 ± 6.3 mm^2^) (**Figure 7B**). The 2 ×IC_90_ dose of SKKUCS resulted in a even smaller lesion size of 12.0 ± 11.0 mm^2^ (**Figure 7C**). As shown in the animal infection site photographs (**Figure 7D**), SKKUCS inhibited skin lesion formation in a dose-dependent manner. Next, bacterial burden was calculated in the skin and spleen one week after infection. IC_50_ dose of SKKUCS reduced the bacterial burden in skin lesions by 55.6% (5.4 × 10^7^ CFU/mL), whereas the IC_90_ dose of SKKUCS reduced the bacterial burden by 74.4% (3.1 × 10^7^ CFU/mL) compared to that in the control. Furthermore, the 2×IC_90_ dose considerably reduced the bacterial count in skin lesions by 89.4%, with a bacterial load of 1.3 × 10^7^ CFU/mL (**Figure 7E**). IC_90_ dose of SKKUCS reduced the bacterial burden by 55.7% with a bacterial count of 2.2 × 10^4^ CFU/mL, whereas the 2xIC_90_ dose of SKKUCS reduced the bacterial burden by 73.2% (1.3 × 10^4^ CFU/mL) in the spleen compared to that in the vehicle-treated animals (**Figure 7F**).

## 4 Discussion

The initial screening revealed that SKKUCS markedly inhibited the SaeRS-GFP reporter strain. SKKUCS was the most effective inhibitor of SaeRS-GFP reporter among all the tested small molecules, without any significant reduction in the growth of *S. aureus* USA300. Moreover, analysis of SKKUCS in HeLa cells revealed no significant cytotoxicity, as its IC_50_ for cell viability was almost four times higher than its calculated IC_90_ for *S. aureus*. These findings highlight the potential of SKKUCS as a promising lead candidate.

Since *S. aureus* relies on the coordinated expression of several virulence factors for the development and progression of infections, we further investigated the effect of SKKUCS on various prominent virulence factors. Previous studies have suggested that virulence genes such as *aur*, *lukS, chp, hla*, and ϒ hemolysin (*hlgA* and *hlgC*) have a direct SaeR-binding site in their promoter regions and are tightly controlled by the SaeRS TCS (Rooijakkers et al., 2006; Cassat et al., 2013; Liu et al., 2016). *S. aureus* relies on the coordinated expression of several virulence factors for the development and progression of infections. Our gene expression analysis revealed that SKKUCS inhibited the expression of genes associated with the *sae* and *agr* loci. Interaction between the *sae* and *agr* loci remains ambiguous. However, previous studies have suggested that these loci function independently (Liu et al., 2016), indicating that SKKUCS potentially interacts with both global regulators. By inhibiting the expression of both *agr* and *sae* regulators, SKKUCS can disrupt the expression of various virulence factors, thereby attenuating the pathogenicity of *S. aureus*. Therefore, targeting multiple loci can aid in the development of potent antivirulence strategies. Furthermore, previous studies showed that interference with global regulators such as *agr* does not exert any selective pressure and, therefore, does not induce resistance against antivirulence agents (Sully et al., 2014).

We further examined the inhibition of pore formation by SKKUCS. The results suggested that SKKUCS may prevent the influx of α-hemolysin and protect the membrane integrity of target cells by inhibiting hemolysin-mediated pore formation in host cell membranes. Moreover, SKKUCS improved the macrophage function by enhancing the phagocytosis and overall clearance of *S. aureus*. By enhancing macrophage-based clearance, SKKUCS also reduced the bacterial burden and prevented the spread of *S. aureus* infection.

*In vivo* results revealed that SKKUCS significantly enhanced the clearance of *S. aureus*, probably by promoting immune cell recruitment and reducing the bacterial burden in various organs. Hemolysins aid in the evasion of immune responses and hemolysin-deficient strains are efficiently cleared by immune cells, resulting in decreased *S. aureus* burden, reduced tissue damage, and improved host survival (Haslinger-Löffler et al., 2005; Spaan et al., 2014; Smith et al., 2018). Here, our findings highlight the potency of SKKUCS as an antivirulence agent that can target specific virulence factors. By inhibiting hemolysin production, SKKUCS disrupts the key mechanism used by *S. aureus* to evade host immune responses. Moreover, treatment with the IC_90_ or an equivalent dose of SKKUCS significantly increases the host survival up to 60%.

Intriguingly, the binding affinities to SaeS of all top-scored docking poses of SKKUCS were estimated to be higher than that of the top-scored docking pose of ATP (**Supplementary Table S3**). This computational prediction implied that SKKUCS potentially functioned as an ATP competitor in inhibiting the SaeS activity. Additionally, our results from dermonecrosis infection suggest that SKKUCS efficiently interferes with the virulence gene expression of *S. aureus* USA300 *in vivo*, leading to reduced tissue damage and inflammation. Substantial reduction in the bacterial burden in the lesion site further confirmed the efficacy of SKKUCS in inhibiting *S. aureus* transmission. Notably, *S. aureus* count was significantly reduced in the spleen after treatment with SKKUCS during dermonecrosis. These results confirm the efficacy of SKKUCS in limiting the spread of infections and preventing secondary infections associated with systemic dissemination.

In conclusion, SKKUCS acts as a novel potent therapeutic antivirulence candidate and inhibits the expression of *hla* in multidrug-resistant *S. aureus*. The *sae* locus tightly controls the expression of *hla* and serves as a crucial regulatory system for α-hemolysin production in *S. aureus* (Gudeta et al., 2019). Based on the results obtained from autokinase inhibition assay and molecular docking suggest that SKKUCS inhibits the kinase activity of SaeS and potentially targets the active site of SaeS kinase, possibly inhibiting ATP binding. Nonetheless, further research is required to elucidate the precise mechanism. Disruption of the SaeRS system notably decreases the expression of *hla*. Additionally, SKKUCS may interfere with the downstream transcriptional regulators or effectors that are under the direct control of the SaeRS system, leading to a reduction in hemolysin production. Overall, the efficacy of SKKUCS as an antivirulence agent suggests interference with the *sae* locus as a promising approach to control the virulence of *S. aureus*. Previous studies have also suggested that the sensing domain of SaeS from *S. aureus* is highly conserved across strains, and no homologues are found in other bacterial species (Giraudo et al., 1999; Flack et al., 2014) Thus, SKKUCS has a narrow-spectrum antivirulence function and no activity towards other pathogens. However, further investigations are necessary to elucidate the specific mechanisms by which SKKUCS exerts its inhibitory effects, facilitating the development of antivirulence agents against *S. aureus* and mitigating the associated clinical challenges.

## Data availability statement

The raw data supporting the conclusions of this article will be made available by the authors, without undue reservation.

## Ethics statement

The animal study was approved by Institutional Animal Care and Use Committee. The study was conducted in accordance with the local legislation and institutional requirements.

## Author contributions

KK: Conceptualization, Funding acquisition, Project administration, Resources, Supervision, Writing – review & editing. RA: Data curation, Formal Analysis, Investigation, Methodology, Validation, Writing – original draft, Writing – review & editing. JY: Investigation, Writing – original draft. TK: Methodology, Validation, Writing-review & editing, Data curation, Writing - original draft. TB: Supervision, Writing-review & editing.
