# Supplementary material for: Identification of an antivirulence agent targeting the master regulator of virulence genes in Staphylococcus aureus
Source: Front Cell Infect Microbiol. 2023 Oct 31;13:1268044. doi: 10.3389/fcimb.2023.1268044 (PMC10644738; doi:10.3389/fcimb.2023.1268044)
Supplement: Supplementary file 1 [file DataSheet_1.docx]

***Supplementary Material***

**Identification of an antivirulence agent targeting the master regulator of virulence genes in *Staphylococcus aureus***

# Rekha Arya^1,2^, Truc Kim^1^, Joo Won Youn^1^ Taeok Bae^3^, and Kyeong Kyu Kim^1*^

^1^Department of Precision Medicine, Sungkyunkwan University School of Medicine, Institute of Antibacterial Resistance Research and Therapeutics, Suwon 16419, Korea.

^2^Department of Orthopedic Surgery, University of Pittsburgh School of Medicine, Pittsburgh, PA, USA.

^3^Department of Microbiology and Immunology, Indiana University School of Medicine-Northwest, Gary, Indiana, USA.

*** Correspondence:**

Kyeong Kyu Kim
kyeongkyu@skku.edu (K. K. Kim)

**Supplementary Figure S1.** The effect of SKKUCS at various concentrations (1µM-120µM) on bacterial growth in USA300 was observed over 12 hours. Results represent the mean ±SEM.


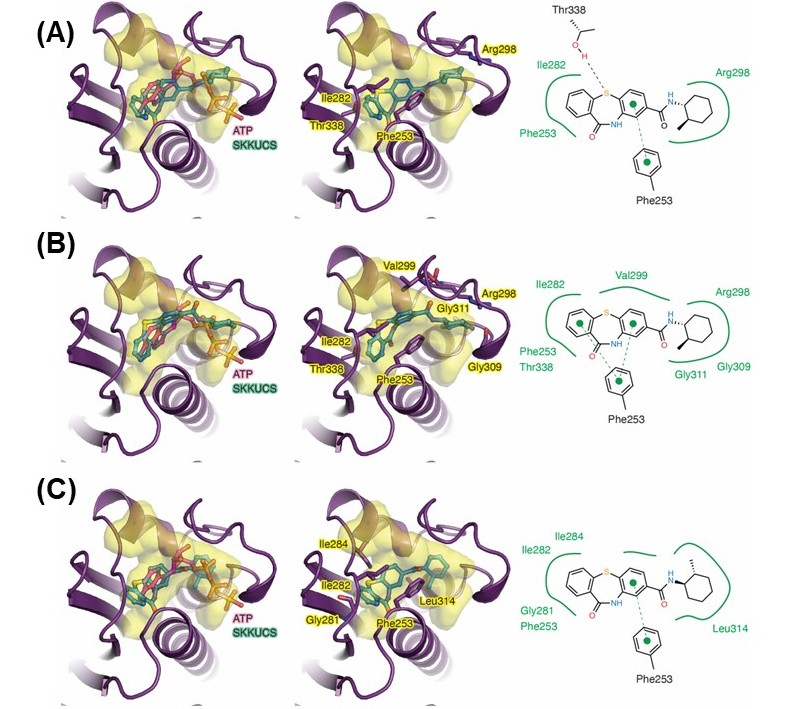


**Supplementary Figure S2. Molecular docking models of SKKUCS in complexes with SaeS.** The chemical SKKUCS was docked into the Alpha Fold structural model for the kinase domain of *S. aureus* SaeS using GNINA (1), and the three top-scored docking poses of SKKUCS within SaeS were individually and respectively presented in (A), (B), and (C). (*Left*) Superimposition of the docking poses of SKKUCS and ATP molecules in the ATP-binding pocket of SaeS. (*Middle*) Predicted residues involved in the accommodation of SKKUCS in the ATP-binding pocket of SaeS. In the *left* and *middle* panels, the ATP-binding domain of SaeS is shown as a cartoon representation. The protein cavity accommodating ATP was calculated using PyVOL (2) and is represented as a transparent surface. The docked SKKUCS and residues involved in SKKUCS binding are shown as stick models. Figures in these panels were prepared using PyMOL (http://www.pymol.org). (*Right*) Two-dimensional schematic diagram illustrating interactions of SaeS and docking poses of SKKUCS. Hydrophobic contacts between protein and chemical are highlighted in green. In (A), a hydrogen bond between the hydroxyl oxygen atom of SaeS Thr338 and the sulfur atom of SKKUCS is drawn as a black dashed line. Figures were redrawn from those generated by PoseView of Proteins*Plus* (3).

**Supplementary Figure S3. Quantitative image analysis of live/dead HeLa cells.** The HeLa cells were infected with USA300 and treated with various doses of SKKUCS. 10x confocal images were obtained, and the percentage of dead cells was quantitatively counted. All experiments were performed in triplicate, and statistical significance was measured using an unpaired two-tailed Student’s t-test. *p < 0.05; **p < 0.01; ***p < 0.001; ns, not significant.

**Supplementary Table S1. Bacterial strains and plasmids used in this study.**

| Strain or Plasmid | Relevant characteristic | Origin |
| --- | --- | --- |
| *E. coli* | | |
| DH5α | Plasmid-free, restriction deficient | New England Biolabs |
| BL21(DE3) | Recombinant protein expression strain | New England Biolabs |
| *S. aureus* | | |
| USA300 | USA300-0114 | (4) |
| USA300-P23 | USA300-0114 without plasmid 2 and 3 | (5) |
| Plasmids | | |
| pYJ335 | An *E. coli-S. aureus* shuttle vector, Ermr | (6) |
| pYJ335-*gfp* | pYJ335 carrying a promoterless *gfp* | (6) |
| pYJ335-P1-*gfp* | pYJ335 carrying the pYJ335 P1promoter-*gfp* fusion | (6) |
| pCL-*Phlam-gfp* | pCL55 carrying *Phlam*-*gfp* fusion | (6) |

**Supplementary Table S2. Primers used in this study.**

| Name | Sequence (5′→ 3′) | Target |
| --- | --- | --- |
| P1969 | GGGGTACCATTGGAAGTGGATAACATGTCAAAAGGAGAA  GAATTATTTAC | *pYJ-gfp* |
| P1747 | ATTGGATTGGAAGTACGGTACCGAGCTCGAATTCACTG | *pYJ-gfp* |
| P1971 | TACTTCCAATCCAATGTCATCATTGGTGGTATTATGTTG | *P1* |
| P1972 | TTATCCACTTCCAATGGCTAACTCCTCATTTCTTCAATT | *P1* |
| P1992 | TTATCCACTTCCAATGattacaatataaaaatacaaatatcttag | *Phla* |
| P1993 | TACTTCCAATCCAATG TTAATATATAGTTAATTTTTATTTAATAG | *Phla* |
| P0011 | GCG CTG CAT TAG CTA GTT GGT  TGG CCG ATC ACC CTC TCA | *16s* |
| P0012 | CAA CAA CAC TAT TGC TAG GTT CCA TAT T  CCT GTT TTT ACT GTA GTA TTG CTT CCA | *α-hemolysin* |
| P0013 | TGT CTG CGT GTA CTT TCA CTT C  AAG AGT GAT GCG GTC AAA GC | *Aureolysin* |
| P0014 | ATC AAT CGG AGG CAG TGG C  GCA GAT ACT TGA CCA TTC GGT G | *ϒ-hemolysin A* |
| P0015 | \| CTA TCA CAC AGA CAA GAT GGC G \| \| --- \| \| CCC AGT AGA AGC CAT TCC AAC \| | *ϒ-hemolysin B* |
| P0016 | \| CTAATCCAGAACCACCGTTT \| \| --- \| \| GCGATGAAGGTATTGGCATTATAC \| | *saeS* |
| P0017 | \| CGTAGTCAACCATTGCGATTTC \| \| --- \| \| CGGTGAAACTGTTGAAGGTAAAG \| | *saeP* |
| P0018 | \| CTCCGAGTGGGACAACAATATC \| \| --- \| \| GTTTAGTACCAGTCATCGCTAACA \| | *saeQ* |
| P0019 | \| CCGCTAGTTGTCGTTGTTACT \| \| --- \| \| CCCACTTACTGATCGTGGATG \| | *saeR* |
| P0020 | \| ACGTGGCAGTAATTCAGTGT \| \| --- \| \| ATGGGCAATGAGTCTGTGAG \| | *agrA* |
| P0021 | \| GACCAGTTTGCCACGTATCT \| \| --- \| \| GCTAAGACCTGCATCCCTAATC \| | *agrB* |
| P0022 | \| GCTGATGATATACCACGCATTC \| \| --- \| \| GACCTAAACCACGACCTTCA \| | *agrC* |
| P0023 | \| AAAACATTGGTAACATCGCAGC \| \| --- \| \| TCTTTTGGTACTTCAACTTCATCCA \| | *agrD* |
| P0024 | \| AGTGACAACGGCGTCATTAG \| \| --- \| \| GCATCTGAACCGATGAAATGTG \| | *Cap8* |
| P0025 | \| TACCCGTTTCCACTTTCG C \| \| --- \| \| GGCTACACAAAATCAAGTCGC \| | *fnbA* |
| P0026 | \| GTGTTGATTGTGATGGTTGCTC \| \| --- \| \| GTAGAGGAAAGTGGGAGTTCAG \| | *fnbB* |
| P0027 | \| GTCTGGAACAAAATAGTCTCTCGG \| \| --- \| \| GGTCCATCAACAGGAGGTAATG \| | *lukS* |
| P0028 | \| ACACACCATCATTCAGCGA \| \| --- \| \| AGCGTT GTAGGAAGACCAC \| | *chp* |
| P0029 | \| TAATGTAGATTGGGCAATTACA \| \| --- \| \| ATGCTTTAATTCAGTTAGAAGC \| | *coa* |

**Supplementary Table S3. Calculated binding affinities to SaeS of GNINA-derived docking poses of ATP and SKKUCS.**

| **Pose** | **GNINA** | | **HAC-Net** | | **SKKUCS - ATP**  **superimposition** |
| --- | --- | --- | --- | --- | --- |
|  | **pK_d_** *^a^* | **K_d_ (μM)** | **pK_d_** *^a^* | **K_d_ ( μM)** |  |
| ATP | 5.501 | 3.16 | 6.146 | 0.71 | 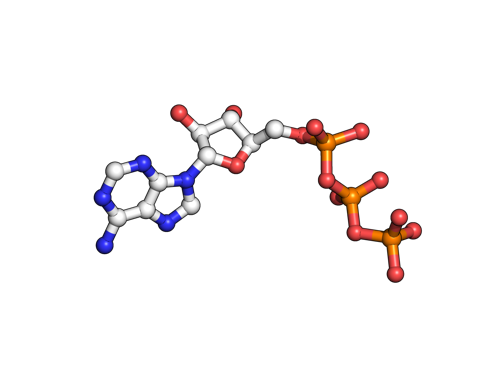 |
| SKKUCS1 | 6.064 | 0.86 | 7.322 | 0.05 | 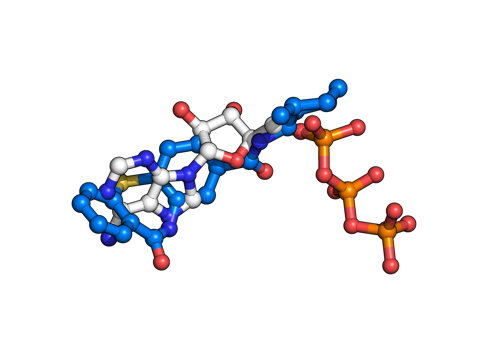 |
| SKKUCS2 | 6.067 | 0.86 | 6.520 | 0.30 | 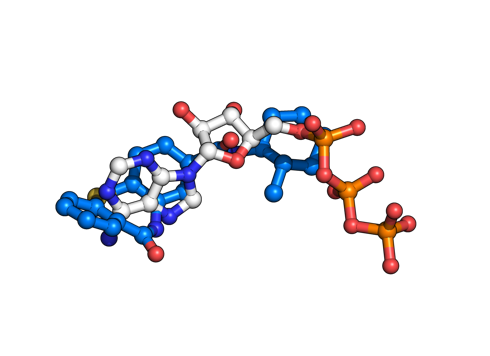 |
| SKKUCS3 | 5.931 | 1.17 | 6.228 | 0.59 | 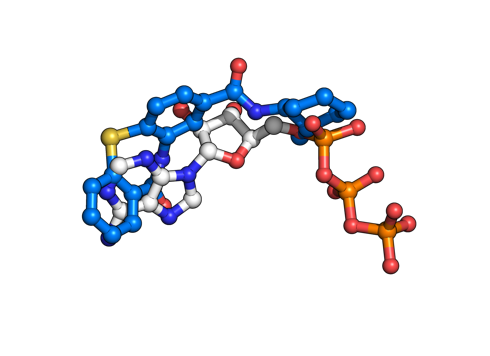 |

*^a^* pK_d_ = −logK_d_

**References:**

1. McNutt AT, Francoeur P, Aggarwal R, Masuda T, Meli R, Ragoza M, et al. GNINA 1.0: molecular docking with deep learning. J Cheminform. 2021;13(1):43.

2. Smith RHB, Dar AC, Schlessinger A. PyVOL: a PyMOL plugin for visualization, comparison, and volume calculation of drug-binding sites. bioRxiv. 2019:816702.

3. Schoning-Stierand K, Diedrich K, Ehrt C, Flachsenberg F, Graef J, Sieg J, et al. ProteinsPlus: a comprehensive collection of web-based molecular modeling tools. Nucleic Acids Res. 2022;50(W1):W611-W5.

4. Baker S, Thomson N, Weill F-X, Holt KEJS. Genomic insights into the emergence and spread of antimicrobial-resistant bacterial pathogens. 2018;360(6390):733-8.

5. Tenover FC, McDougal LK, Goering RV, Killgore G, Projan SJ, Patel JB, et al. Characterization of a Strain of Community-AssociatedMethicillin-Resistant Staphylococcus Aureus WidelyDisseminated in the UnitedStates. 2006;44(1):108-18.

6. Yeo W-S, Arya R, Kim KK, Jeong H, Cho KH, Bae T. The FDA-approved anti-cancer drugs, streptozotocin and floxuridine, reduce the virulence of Staphylococcus aureus. Scientific reports. 2018;8(1):1-10.
